# Information diversity in individual auditory cortical neurons is associated with functionally distinct coordinated neuronal ensembles

**DOI:** 10.1038/s41598-021-83565-7

**Published:** 2021-02-18

**Authors:** Jermyn Z. See, Natsumi Y. Homma, Craig A. Atencio, Vikaas S. Sohal, Christoph E. Schreiner

**Affiliations:** 1 Weill Institute for Neuroscience, Kavli Institute for Fundamental Neuroscience, and Sloan-Swartz Center for Theoretical Neurobiology, University of California, San Francisco, 675 Nelson Rising Lane, San Francisco, CA 94158-0444 USA; 2 Department of Otolaryngology-Head and Neck Surgery, Coleman Memorial Laboratory, University of Caliornia, San Francisco, USA; 3 Department of Psychiatry, University of California, San Francisco, USA

**Keywords:** Neuroscience, Auditory system, Computational neuroscience, Sensory processing

## Abstract

Neuronal activity in auditory cortex is often highly synchronous between neighboring neurons. Such coordinated activity is thought to be crucial for information processing. We determined the functional properties of coordinated neuronal ensembles (cNEs) within primary auditory cortical (AI) columns relative to the contributing neurons. Nearly half of AI cNEs showed robust spectro-temporal receptive fields whereas the remaining cNEs showed little or no acoustic feature selectivity. cNEs can therefore capture either specific, time-locked information of spectro-temporal stimulus features or reflect stimulus-unspecific, less-time specific processing aspects. By contrast, we show that individual neurons can represent both of those aspects through membership in multiple cNEs with either high or absent feature selectivity. These associations produce functionally heterogeneous spikes identifiable by instantaneous association with different cNEs. This demonstrates that single neuron spike trains can sequentially convey multiple aspects that contribute to cortical processing, including stimulus-specific and unspecific information.

## Introduction

Neurons in the cortex have been shown to be highly interconnected^1^. As such, the activity of any individual neuron is strongly correlated with some of its neighbors, and this coordinated activity is thought to underlie information processing and transmission throughout the cortex^2–6^. This is due in part to the robustness of information encoding in neuronal ensembles over single neurons^7^, and in part to the increased likelihood of information transmission by synchronously firing neurons^8–11^.

In the primary auditory cortex (AI), the activity of coordinated neuronal ensembles (cNEs), defined as groups of neurons with reliable and precise synchrony, are stable functional constructs^12–16^. They are present in both spontaneous and evoked activity, and can encode more information about the auditory stimulus than single neurons or random groups of simultaneously recorded neurons, suggesting that they may represent a principal unit of information processing^7^.

Meanwhile, other studies in AI have shown the existence of populations of neurons that do not appear to encode specific spectral or temporal features of an auditory stimulus^17,18^, but instead encode other aspects of auditory processing, such as the choice of an animal in a discrimination task^19,20^, the categorization of a sound based on an animal’s percept^2^, or associated multisensory or cognitive cues^21,22^. Our goal in this study was to determine the relationship of the functional properties of individual neurons and their associated cNEs to assess the degree of functional conformity between single neuron and ensemble properties.

We first characterized the functional properties of cNEs in rat AI columns, while presenting dynamic broadband stimuli. We obtained cNEs using dimensionality reduction techniques and identified cNE events^7^, using them to assess their stimulus-related information. We find that there are different functional categories of cNEs—some can be explicitly related to spectro-temporal stimulus information, while others seem agnostic or only indirectly related to the presented stimuli. We further explored the relationship of cNE functional properties to the individual neurons that contribute to one or more cNE. By grouping individual spikes of single neurons according to instantaneous association with the activities of different cNEs, we find that individual AI neurons can produce functionally heterogeneous sets of spikes that encode either explicit spectro-temporal feature information, or other types of information. By linking individual neuronal events to the activity of local networks, we were able to perform a more refined analysis of cortical processing principles.

## Results

### cNE activity can be stimulus-specific or stimulus-unspecific

Our main goal was to determine how well stimulus-related properties of cNEs and their contributing single neurons are matched. We sampled the activity of populations of neurons in cortical columns of rat primary auditory cortex (AI) with high-density recording arrays across the entire cortical depth. Sites had well-defined tuning curves when presented with pure tones. Neuronal recordings were made from anesthetized rats while a dynamic broadband stimulus (dynamic moving ripple—DMR)^23^ was presented. Spectro-temporal receptive fields (STRFs) of well-isolated neurons were estimated via spike-triggered averaging (STA; Supplementary Fig. 1a). Statistical significance of STRFs was determined via a reliability index metric (RI; Supplementary Fig. 1b, c)^24^. RI is a proxy for the homogeneity of neuronal spikes with respect to the presented stimulus.

**Figure 1:**
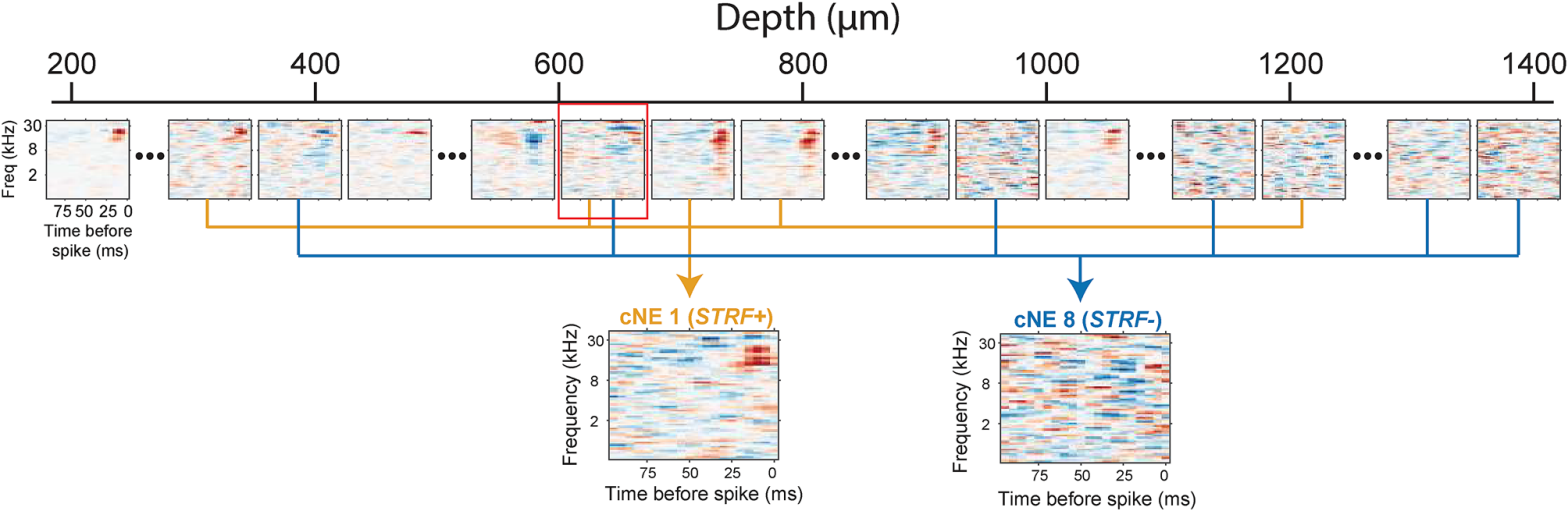
Example penetration with two sample cNEs. *STRF*+: neurons or cNEs with significant STRFs. *STRF−*: neurons or cNEs with non-significant STRFs. Depth of some neurons and their respective STRFs are shown. Two example cNEs are represented. cNE 1 (orange, *STRF*+) is mostly made up of *STRF*+ neuronal members. cNE 8 (blue, *STRF−*) is mostly made up of *STRF−* neuronal members. One neuron, highlighted with a red square at a depth of approximately 600 µm, is part of both cNE 1 and cNE 8.

cNEs for a columnar recording were extracted by applying principal and independent component analyses to the recorded spike trains to identify groups of neurons that fired synchronously (see “Methods”; Supplementary Fig. 1d)^7,25^. The cNE event train is analogous to a neuronal spike train and can be used to calculate an STRF (Supplementary Fig. 1a–c). An example columnar penetration reveals feature-selective STRFs for some neurons and one cNE as well as several feature-unspecific neurons and one cNE (Fig. 1). Neurons or cNEs with statistically significant STRFs are labeled *STRF*+ (stimulus-specific), while neurons or cNEs without a significant STRF are labeled *STRF−*(stimulus-unspecific; Fig. 1).

We found that *STRF*+ neurons that contribute to *STRF*+ cNEs had similar, but not necessarily identical, STRFs as the cNE that they contribute to (see cNE1, Fig. 1). *STRF− *cNEs (see cNE 8, Fig. 1) are made up of neurons that, across all spikes, can fire synchronously and above chance with high temporal precision, and their activity cannot be trivially attributed to “noise events”. Thus, the absence (*STRF−*) or presence (*STRF*+) of a statistically significant STRF in a cNE is not predicated by the degree of synchrony between member neurons in a cNE. Both types of cNEs are equivalent in their defining properties and inclusion criteria.

Previously we showed that *STRF*+ cNEs are stable across spontaneous and stimulus-evoked epochs, and that they are not a trivial result of stimulus synchronization^7^. Here, we asked if there was any difference in stability across spontaneous and evoked epochs between *STRF*+ and *STRF−* cNEs. For each isolated stimulus-evoked cNE, we searched for a significant match amongst the contiguously recorded spontaneous cNEs (Supplementary Fig. 2a–d). Considering only significant matches, we z-transformed the correlation values between matched cNEs based on a null shuffled distribution. We found no significant difference in composition and stability between *STRF*+ and *STRF−* cNEs (Supplementary Fig. 2e; Mann–Whitney U test, p = 0.13). This reinforces the notion that *STRF−* cNEs are as stable and likely biologically relevant as *STRF*+ cNEs and cannot be attributed as “noise”, and also re-emphasizes the fact that *STRF*+ cNEs cannot be trivially attributed to receptive field overlaps of the contributing neurons.

### Classification of cNEs based on STRF significance

**Figure 2:**
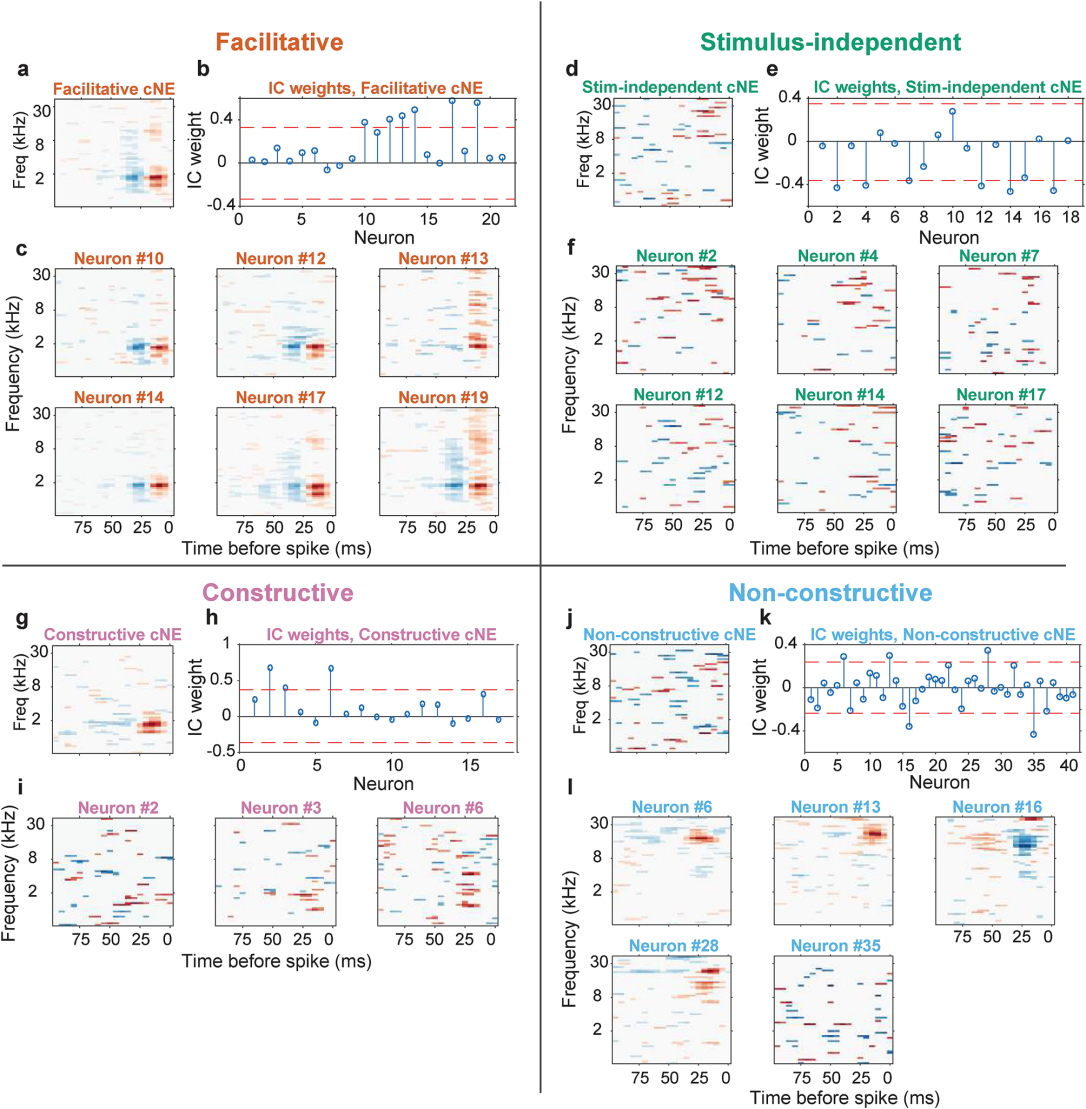
STRFs of cNEs and member neurons. (**a**–**c**) Facilitative cNE is *STRF*+ and most of its member neurons are also *STRF*+. (**d**–**f**) Stimulus-independent cNE is *STRF−* and most of its member neurons are also *STRF−*. (**g**–**i**) Constructive cNE is *STRF*+ but most of its member neurons are *STRF−*. (**j**–**l**) Non-constructive cNE is *STRF−* but most of its member neurons are *STRF*+. (**a**, **d**, **g**, **j**) STRFs of the cNEs. (**b**, **e**, **h**, **k**) IC weights for each cNE. The magnitude of the IC weights represents the contribution of each neuron to the cNE. Red dashed lines: thresholds that determine if a neuron is a member of the cNE. (**c**, **f**, **i**, **l**) STRFs of member neurons.

Based on the significance of neuronal and cNE STRFs, we observed four broad categories of cNEs. (1) *Facilitative cNEs* are cooperative *STRF*+ cNEs, and a majority of their member neurons are also *STRF*+ (Fig. 2a-c). (2) *Stimulus-independent cNEs* are *STRF−* cNEs, with member neurons that are generally *STRF−* but can contain subsets of spikes with high synchrony to other cNE members (Fig. 2d–f). Two less common types of cNEs were seen: (3) *constructive cNEs* are *STRF*+ while most of its member neurons are *STRF−* (Fig. 2g–i) and (4) *non-constructive cNEs* are *STRF−* while most of its member neurons are *STRF*+ (Fig. 2j–l).

**Figure 3:**
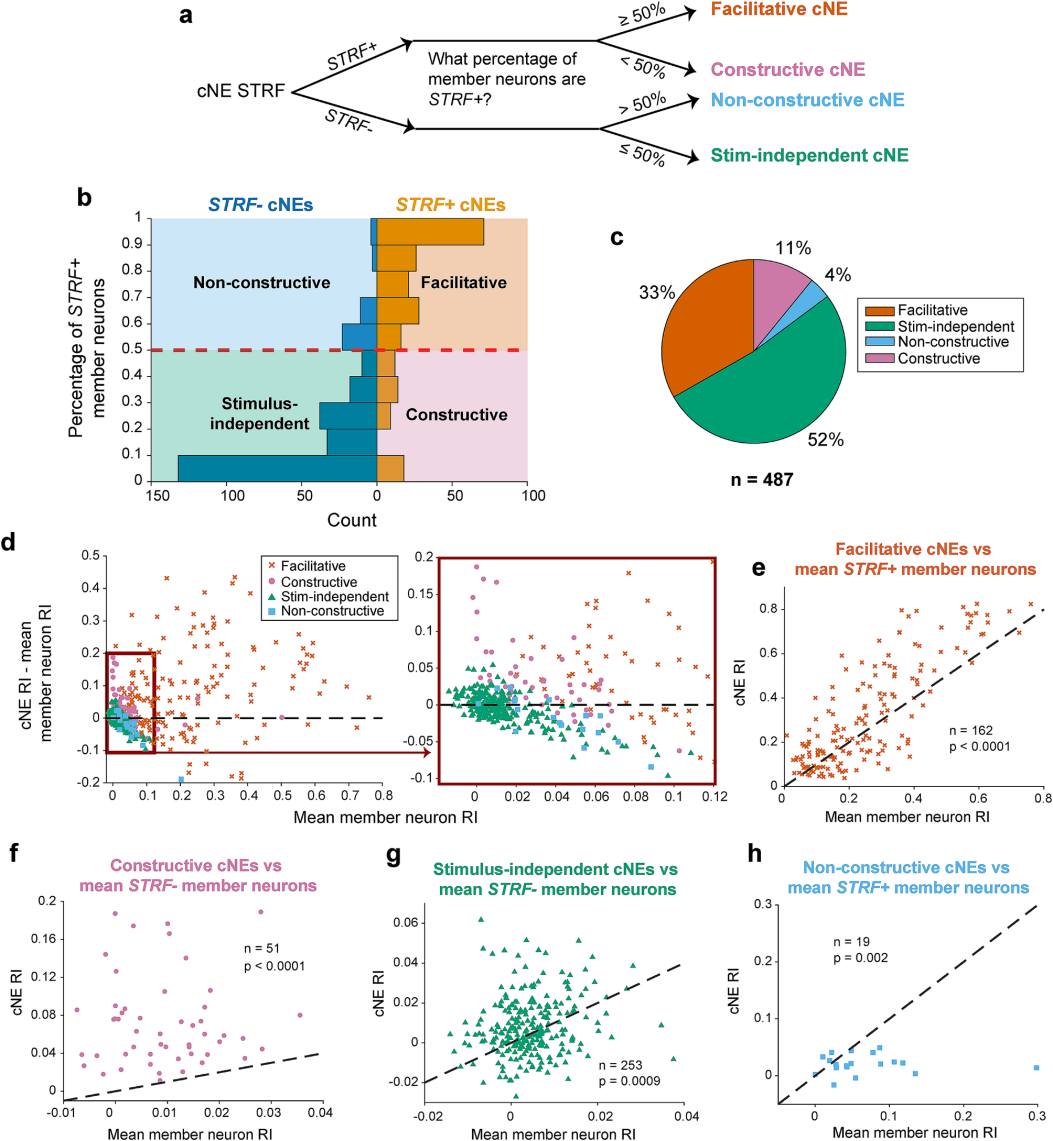
Categorization of cNE groups. (**a**) Flow chart illustrating how cNEs were categorized. (**b**) Histogram of cNE categories. (**c**) Pie chart illustrating the breakdown in percentages of the four different categories of cNEs. (**d**) (Left) Mean of the difference between cNE Reliability Index (RI) and member neuron RI vs mean RI of all member neurons. Only facilitative cNEs exist outside the red square, apart from a few exceptions. (Right) Enlarged version of left graph, indicated by the red square. Constructive cNEs mostly fall above the black dashed line (i.e., cNE RI > member neuron RI), stimulus-independent cNEs fall on either side of the black dashed line and are mostly clustered at low values around 0, while non-constructive cNEs cluster below the black dashed line. The four different groups form different clusters although with some overlap. (**e**) RI between facilitative cNEs and the mean RI of *STRF*+ member neurons. ≈ 67% of cNEs had higher RIs than that of their member neurons. (**f**) Same as (**e**), but for stimulus-independent cNEs and *STRF−* neurons. ≈ 56% of cNEs had higher RIs than that of their *STRF−* member neurons. (**g**) Same as (**e**), but for constructive cNEs and *STRF−* member neurons. 100% of cNEs had higher RIs than that of their member neurons. (**h**) Same as (**e**), but for non-constructive cNEs and *STRF*+ member neurons. ≈ 21% of cNEs had higher RIs than that of their member neurons. (**e**–**h**) Wilcoxon signed-rank test.

Across all four categories of cNEs (N = 487), stimulus-independent cNEs were most common (52%), followed by facilitative cNEs (33%), constructive cNEs (11%) and non-constructive cNEs (4%; Fig. 3a–c).

We verified the validity of our classification scheme using several quantitative comparisons. Plotting the mean difference between the RIs of cNEs and their member neurons, against the mean RI of member neurons, the groups were well-clustered; facilitative cNE generally had member neurons with high average RI (> 0.1); most constructive cNEs had mean differences > 0 while most non-constructive cNEs had mean difference < 0; and most stimulus-independent cNEs had member neurons with average RI close to 0 (Fig. 3d).

Next, we compared the RI of facilitative cNEs (*STRF*+) against the average RI of *STRF*+ member neurons. Facilitative cNEs had significantly higher RIs than the mean RIs of their member neurons (Fig. 3e; Wilcoxon signed-rank test, p < 0.0001), with ~ 67% of facilitative cNEs having a higher RI than the RI mean of their constituent *STRF*+ member neurons. We found that this increased stimulus-encoding capacity of cNEs was not driven primarily by the member neurons with the strongest STRFs (i.e., neurons with the highest RI in each cNE). We related the RI difference between member neurons and facilitative cNEs with their IC weights. If a high neuronal RI was predictive of a neuron’s contribution to each cNE, then we would expect that those neurons also have high IC weights (Supplementary Fig. 3a). We noticed only a slight trend towards higher RI difference values as IC weight increases accounting only for about 0.02 of the variance (Supplementary Fig. 3b; R^2^ = 0.024). Thus, the encoding of acoustic information by cNEs is not dominated by single member neurons with the most significant STRFs, but appears to reflect coordination across all member neurons.

For stimulus-independent cNEs (*STRF−*) we used this comparison against their *STRF−* member neurons, and both cNE RIs and average neuronal RIs were clustered around 0 (|RI|< 0.06), with ~ 56% of stimulus-independent cNEs having larger RIs than the average RI of their *STRF−* member neurons (Fig. 3f). Constructive cNEs (*STRF*+) were compared against their *STRF−* member neurons, and cNE RIs were significantly higher than the average RIs of their *STRF−* member neurons (Wilcoxon signed-rank test, p < 0.0001), with 100% of constructive cNEs having larger RIs than the mean RIs of their constituent *STRF−* member neurons (Fig. 3g). Non-constructive cNEs (*STRF−*) were compared against their *STRF*+ member neurons, and cNE RIs were significantly lower than that of their member neurons (Wilcoxon signed-rank test, p = 0.002), with only 21% of non-constructive cNEs having larger RIs than the mean RIs of their constituent *STRF*+ member neurons (Fig. 3h). These observations verified that our classification method was robust and functionally consistent.

### Physical and functional properties of cNE categories

**Figure 4:**
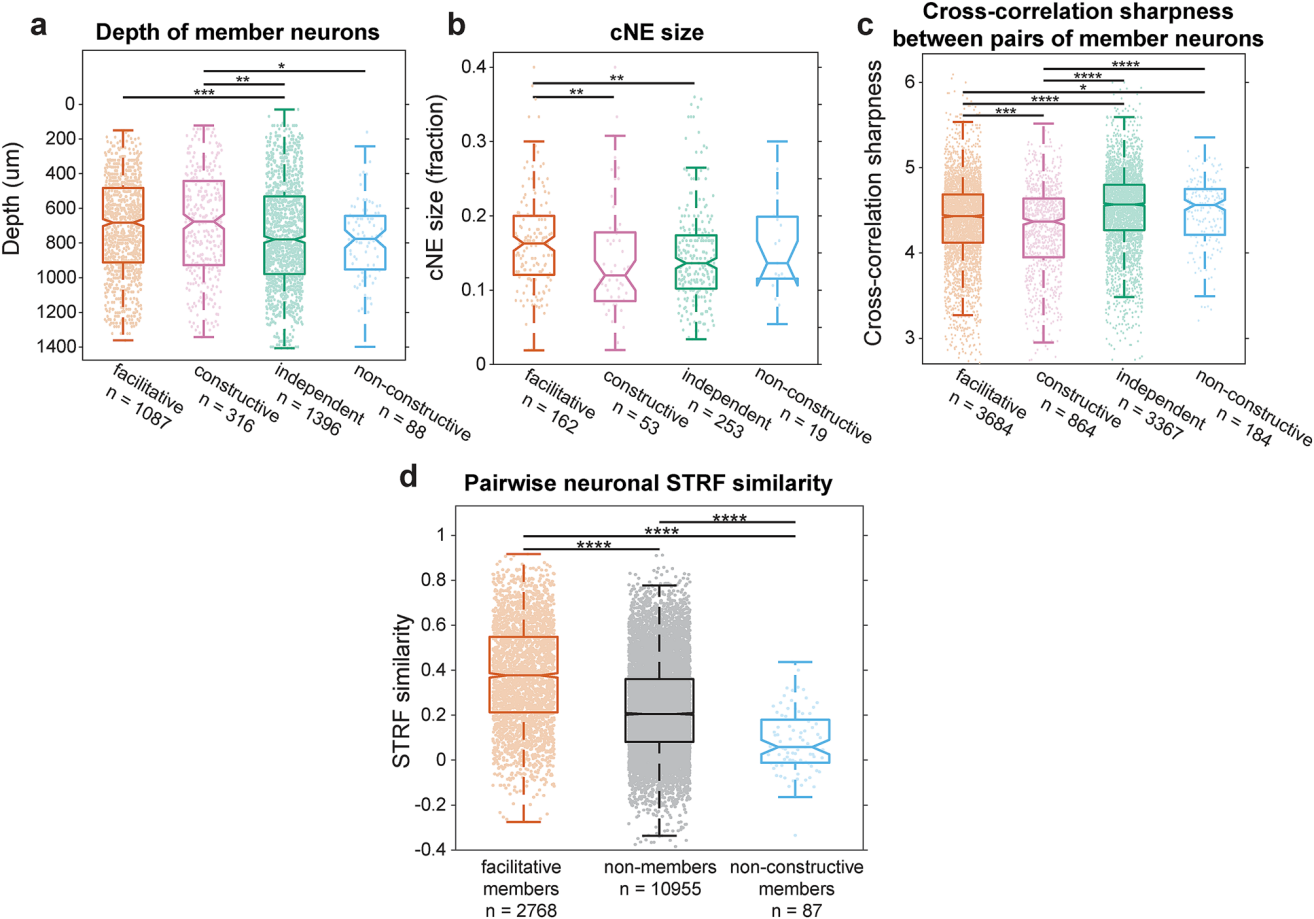
Functional and physical properties of cNE categories. (**a**) Depth of member neurons between different types of cNEs. Constructive and facilitative cNEs were found at a shallower depth than stimulus-independent and non-constructive cNEs. (**b**) cNE size, calculated by the number of neuronal members as a fraction of the total number of neurons recorded. Facilitative cNEs were larger than constructive or stimulus-independent cNEs. (**c**) Cross-correlation sharpness (see “Methods”) between pairs of neurons from the same cNE. Stimulus-independent and non-constructive cNEs had broader cross-correlation functions than facilitative or constructive cNEs. (**d**) STRF similarity between pairs of *STRF*+ neurons within the same facilitative cNE, non-constructive cNEs, and neurons that were not part of the same cNE but recorded simultaneously. Facilitative cNE members had more similar STRFs than that of non-members while non-constructive cNE members had less similar STRFs that that of non-members. *p < 0.05, **p < 0.01, ***p < 0.001, ****p < 0.0001; Kruskal–Wallis test with Bonferroni correction.

The four categories of cNEs had distinct physical and functional properties. Stimulus-independent and non-constructive cNEs (i.e., *STRF−* cNEs) had member neurons that were located slightly deeper in the cortical column, while facilitative and constructive cNE member neurons were generally found at slightly shallower depths (*STRF*+ cNEs; Fig. 4a, Supplementary Fig. 4c). Facilitative cNEs contained significantly higher numbers of member neurons than constructive or stimulus-independent cNEs (Fig. 4b). All *STRF*+ cNEs tended to be larger in comparison to *STRF−* cNEs (Supplementary Fig. 4d). Constructive cNEs had neuronal member pairs with the sharpest cross-correlation functions, followed by facilitative cNEs, while the stimulus-independent and non-constructive cNEs had broader cross-correlation functions (Fig. 4c, Supplementary Fig. 4e).

For facilitative and non-constructive cNEs that had a majority of *STRF*+ member neurons, we tested whether cNEs are dominated by neurons with high STRF similarity. We found that STRF similarity between pairs of neuronal members in facilitative cNEs were higher than that of pairs of simultaneously-recorded columnar neurons that were not contributing to the same cNE (Fig. 4d). The opposite was seen for non-constructive cNEs; pairs of neuronal members in non-constructive cNEs had lower STRF similarity than pairs of neurons not in the same cNE (Fig. 4d). Neuronal members in facilitative cNEs having more similar STRFs than other simultaneously recorded neurons supporting the idea of more selective spectro-temporal encoding by cNEs.

### Functional considerations for *STRF−* cNEs

To establish some functional aspects and constraints of *STRF−* cNEs, we performed three analyses to test whether their activity is (1) modulated by the presence of a stimulus, (2) correlated to UP states, and (3) responsive to acoustic parameters without tight temporal alignment.

**Figure 5:**
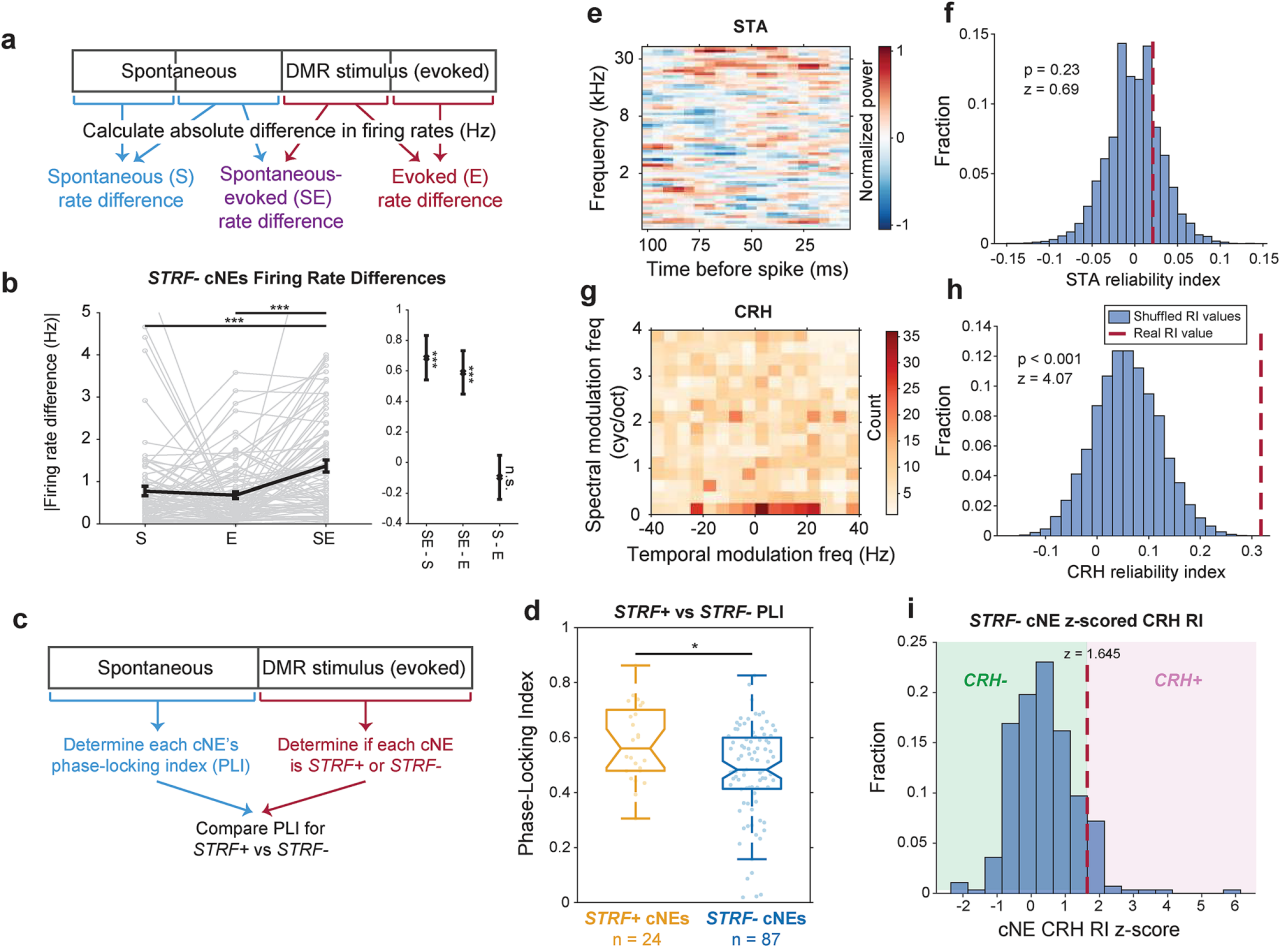
Functional properties of *STRF−* cNEs. (**a**) Illustration of how rate differences between contiguous spontaneous and evoked epochs were calculated. Both epochs were split into two, and the absolute rate differences between contiguous spontaneous (S) and evoked (E) epochs were compared with the rate differences between contiguous spontaneous and evoked epochs (SE). (**b**) Absolute firing rate differences between spontaneous and evoked (SE) epochs were higher than that of contiguous epochs of the same time (S or E). (left) Raw absolute firing rate differences. (right) Paired differences in absolute firing rate differences. (**c**) Illustration of how phase-locking index (PLI), a proxy for the extent of cNE coupling to UP states, was calculated (also see Supplementary Fig. 5 and “Methods”). (**d**) *STRF*+ cNEs had higher PLIs than *STRF−* cNEs, suggesting that *STRF−* cNEs are not trivial results of UP states. (**e**–**h**) Example *STRF−* cNE with a significant conditioned response histogram (CRH). (**e**) STA of example *STRF−* cNE. (**f**) RI of STA for example *STRF−* cNE. (**g**) CRH of example *STRF−* cNE. (**h**) RI of CRH for example *STRF−* cNE. (**i**) Histogram of z-scored CRH RI for *STRF−* cNEs. *STRF−* cNEs with a significant CRH only make up about 9% of *STRF−* cNEs, and cannot explain the large majority of *STRF−* cNEs we observed.

First, to show that *STRF−* cNEs are modulated by the presence of a stimulus, we determined the absolute change in firing rates between two epochs of either spontaneous (S), evoked (E), or between spontaneous and evoked (SE) epochs, thus capturing both rate increases and decreases (Fig. 5a). *STRF−* cNEs had higher firing rate differences between spontaneous and evoked epochs (SE) than between epochs of the same type (S or E; Fig. 5b). This suggests that activity of *STRF−* cNEs is modulated by broadband stimuli.

Next, we show that *STRF−* cNEs are not trivial consequences of UP and DOWN states that are commonly present in the cortex of sleeping or anesthetized animals^26,27^. We used a phase-locking index (PLI) to determine a cNE’s synchronization to the state phase. Because our broadband stimulus desynchronizes cortical activity^28^, we limited PLI analysis to UP states in spontaneous epochs, while classifying *STRF*+ and *STRF−* cNEs from driven epochs (Fig. 5c). We constructed spike rasters and PSTHs of spontaneous multi-unit activity (MUA) across all channels of the electrode array (Supplementary Fig. 5a,c; dark green solid line) and determined the oscillation period and instantaneous phase between the two states (see “Methods”; Supplementary Fig. 5c). Histograms for cNE events relative to UP phases (Supplementary Fig. 5d) show that *STRF*+ cNEs have a higher PLI than *STRF−* cNEs, suggesting that *STRF−* cNEs are aligned less precisely to the UP state than *STRF*+ cNEs in spontaneous activity (Fig. 5d), and their activities cannot be attributed to the presence of an UP state alone.

Finally, we assessed whether *STRF−* cNEs could be explained as stimulus-driven but with insufficient time-locking between spectro-temporal content and activity (Fig. 5e–i). We constructed conditioned response histograms (CRHs)^23^, based on the instantaneous spectral and temporal modulation parameters of DMR stimuli, for all cNE events (e.g., Fig. 5g) and determined significance using RI, identical to how *STRF*+ cNEs were defined (Fig. 5f,h). Since DMRs change their spectro-temporal content relatively slowly, a significant timing mismatch between cNE events and the instantaneous spectro-temporal configuration has a negligible effect on the construction of CRHs in contrast to a large effect on the STA. Calculating the RI for CRHs revealed that only ~ 9% of *STRF−* cNEs had significant CRHs, (p < 0.05 or z > 1.645; Fig. 5i; red dashed line), suggesting that only few *STRF−* cNEs can be accounted for by their temporally loose alignment to spectral or temporal modulation characteristics.

### cNE-associated spiking events have superior stimulus-encoding properties

**Figure 6:**
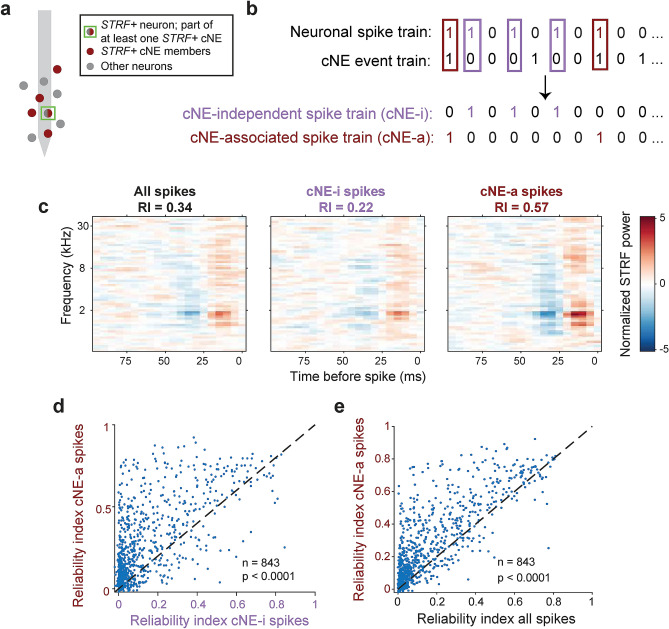
cNE-associated (cNE-a) spikes are more stimulus-dependent than cNE-independent (cNE-i) spikes. (**a**) Schematic of a sample *STRF*+ neuron (green square) relative to the electrode (light gray) that is part of at least one *STRF*+ cNE. This neuron is part red and part gray to illustrate the splitting of its spikes into smaller subsets. (**b**) Illustration of a neuronal spike train split into cNE-i and cNE-a subset spike trains based on coincident activity with cNE events. (**c**) STRFs for the sample neuron’s entire spike train (all spikes), cNE-i and cNE-a subset spike trains. Spike trains were sub-sampled to match the number of spikes across all three types of spike trains. STRF power was also normalized across all three STRFs. The cNE-a STRF had the highest power, followed by the STRF for all spikes and the cNE-i STRF. (**d**) Across the entire cNE population, cNE-a spikes had higher RI than cNE-i spikes (≈ 80% above black dashed unity line). (**e**) cNE-a spikes also had higher RI than all spikes (≈ 79% above black dashed unity line). (**d**, **e**) Paired t-test.

We next wanted to parse the principles by which cNEs enhance auditory information. We know that *STRF*+ neurons respond to the stimulus in a non-linear manner—the more similar the match (i.e. projection value) between the neuron’s STRF and a stimulus segment, the higher the probability of firing past a projection value threshold^29^. Hence, we tested the hypothesis that cNEs select for neuronal member spikes with higher projection values. We divided the spike train of each member neuron of *STRF*+ cNEs into two subsets: the cNE-associated spike train (cNE-a) includes only spikes that correspond to cNE events and the cNE-independent associated spike train (cNE-i) includes the remaining spikes (Fig. 6a,b). After sub-sampling the spike trains to select equal number of spikes, we found that the STRFs corresponding to the cNE-a spikes had the strongest STRF (i.e. highest absolute normalized power), followed by the STRF from all spikes, and then the STRF from cNE-i spikes (Fig. 6c).

Across the entire population of recorded neurons, we compared spikes of *STRF*+ neurons that were part of at least one *STRF*+ cNE. We also took the additional step of requiring that the spike waveforms of cNE-i and cNE-a subsets were similar by comparing the distribution of their spike waveforms (Supplementary Fig. 6). We found that RIs for cNE-a spikes were higher than RIs for cNE-i spikes (paired t-test, p < 0.0001; Fig. 6d) and that they were also significantly higher than that of all spikes in a train (paired t-test, p < 0.0001; Fig. 6e), suggesting that cNE-a spikes correspond to stimulus segments with high projection values and are more reliable in encoding stimulus parameters than cNE-i spikes.

### Spikes of a neuron associated with different cNEs diverge in their stimulus-encoding properties

**Figure 7:**
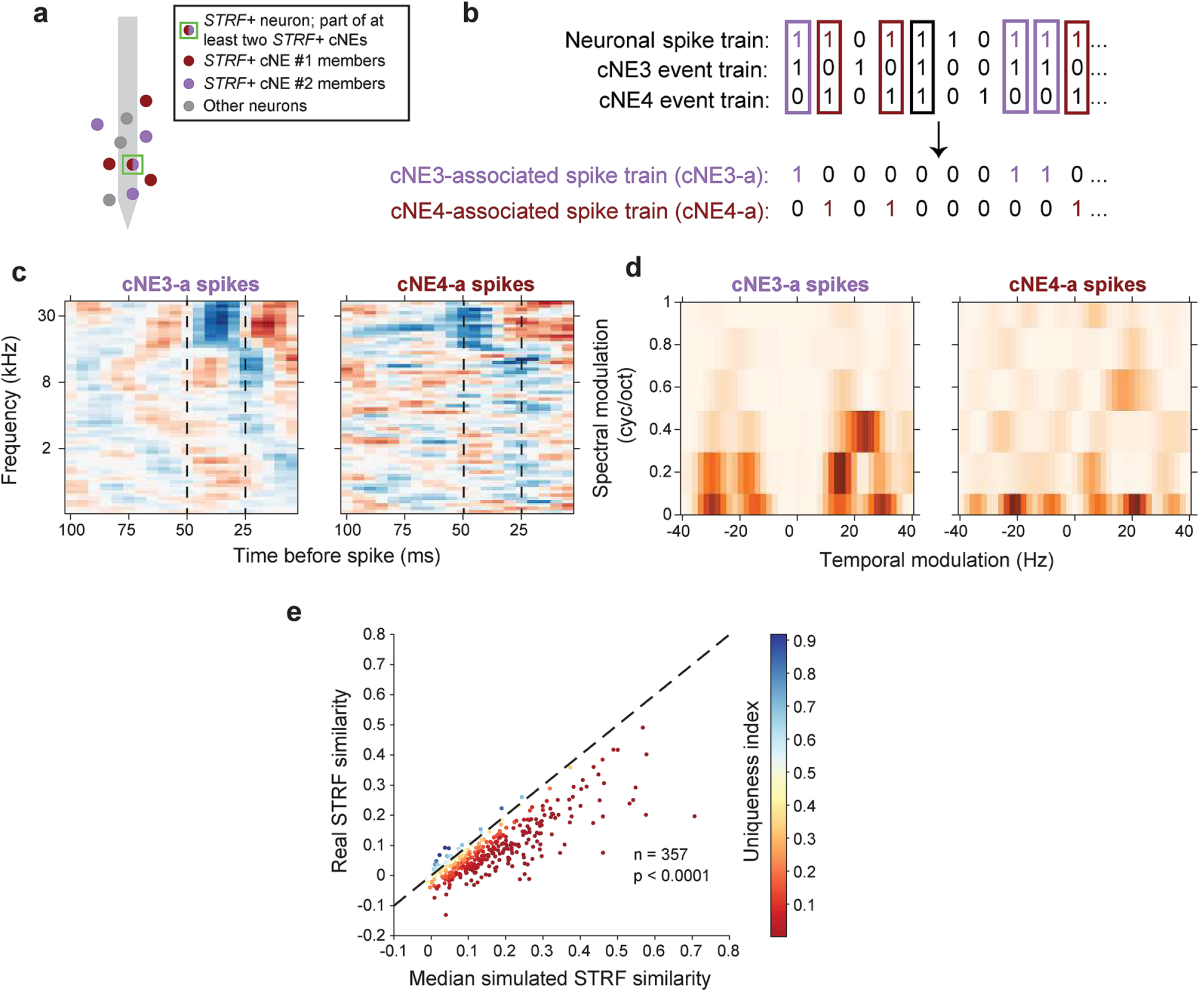
cNE-associated STRFs generated for a neuron participating in two *STRF*+ cNEs are dissimilar. (**a**) Schematic of an *STRF*+ sample neuron (green square) that is part of at least two *STRF*+ cNEs. This neuron is marked part red and part purple to illustrate the splitting of its spikes into smaller subsets. (**b**) Illustration of a neuronal spike train split into two cNE-a subsets. Purple: spikes associated with cNE3; red: spikes associated with cNE4; spikes associated with both cNEs (black rectangle) or neither of the cNEs are omitted. (**c**) Sample STRFs from the two subset spike trains. Vertical black dashed lines demarcate the 25 ms and 50 ms time bins, showing that the STRFs clearly differ in their temporal profile. (**d**) Sample Modulation Transfer Functions from the two subset spike trains. The preferred spectral modulation and temporal modulation frequencies between the two subsets are visually different. (**e**) Real STRF similarity vs median simulated STRF similarity (null distribution; Supplementary Fig. 7a, b) for pairs of cNE-a spike trains across the entire population. Real STRF similarity is significantly lower than the null distribution (≈ 90% below black dashed unity line), implying that spikes associated with different cNEs generated STRFs that were significantly more different than by chance. (**e**) Paired t-test.

A single neuron can be a member of more than one cNE (Fig. 1). Do spikes of a neuron that are associated with the activity of different cNEs encode the same stimulus aspect? We looked at *STRF*+ neurons that were part of two or more *STRF*+ cNEs (“shared neurons”; Fig. 7a). For each “shared neuron”, we subdivided the spike train, each subset containing equal numbers of spikes that were uniquely associated with one of the cNEs (Fig. 7b,c). STRFs from spikes of a neuron associated with distinct cNEs differed in their distribution of excitatory and inhibitory subfields (Fig. 7c). This difference is reflected in each subset’s modulation transfer function^30^ that captures the encoded spectral and temporal modulation preferences. In the example neuron, the spectral and temporal modulation profiles for cNE4-a spikes were lower than that of cNE3-a spikes, despite coming from the same neuron (Fig. 7d).

To quantify STRF differences between two cNE-associated spike trains of a “shared neuron”, we compared the similarity of the two real STRFs against a null distribution derived from combining spikes from each pair of STRFs and calculating the STRF similarity (Supplementary Fig. 7a). A “uniqueness index” was then assigned (see “Methods”, Supplementary Fig. 7b) that indicates whether different cNE-associated STRFs from a neuron were more dissimilar than would be expected by chance. Indeed, across the entire population of “shared neurons”, nearly 50% had significant “uniqueness indices” < 0.05, and ~ 26% had “uniqueness indices” < 0.001 (Supplementary Fig. 7c). The real similarity values for STRFs associated with two different cNEs were also significantly lower than the median of the null distributions (paired t-test, p < 0.0001; Fig. 7e), implying that many “shared neurons” can encode different stimulus parameters when associated with different cNEs.

To ensure that the significant differences we have observed in the STRFs of the cNE-a subsets (Fig. 7b) cannot be trivially attributed to the differences in their cNEs’ STRFs, we compared the STRF similarity between cNEs and neuronal subsets, sub-sampled to give equal numbers of events or spikes (Supplementary Fig. 8a), and found that the neuronal subset STRF similarity was significantly lower than that of the corresponding cNE STRF similarity (paired t-test, p < 0.0001; Supplementary Fig. 8b).

**Figure 8:**
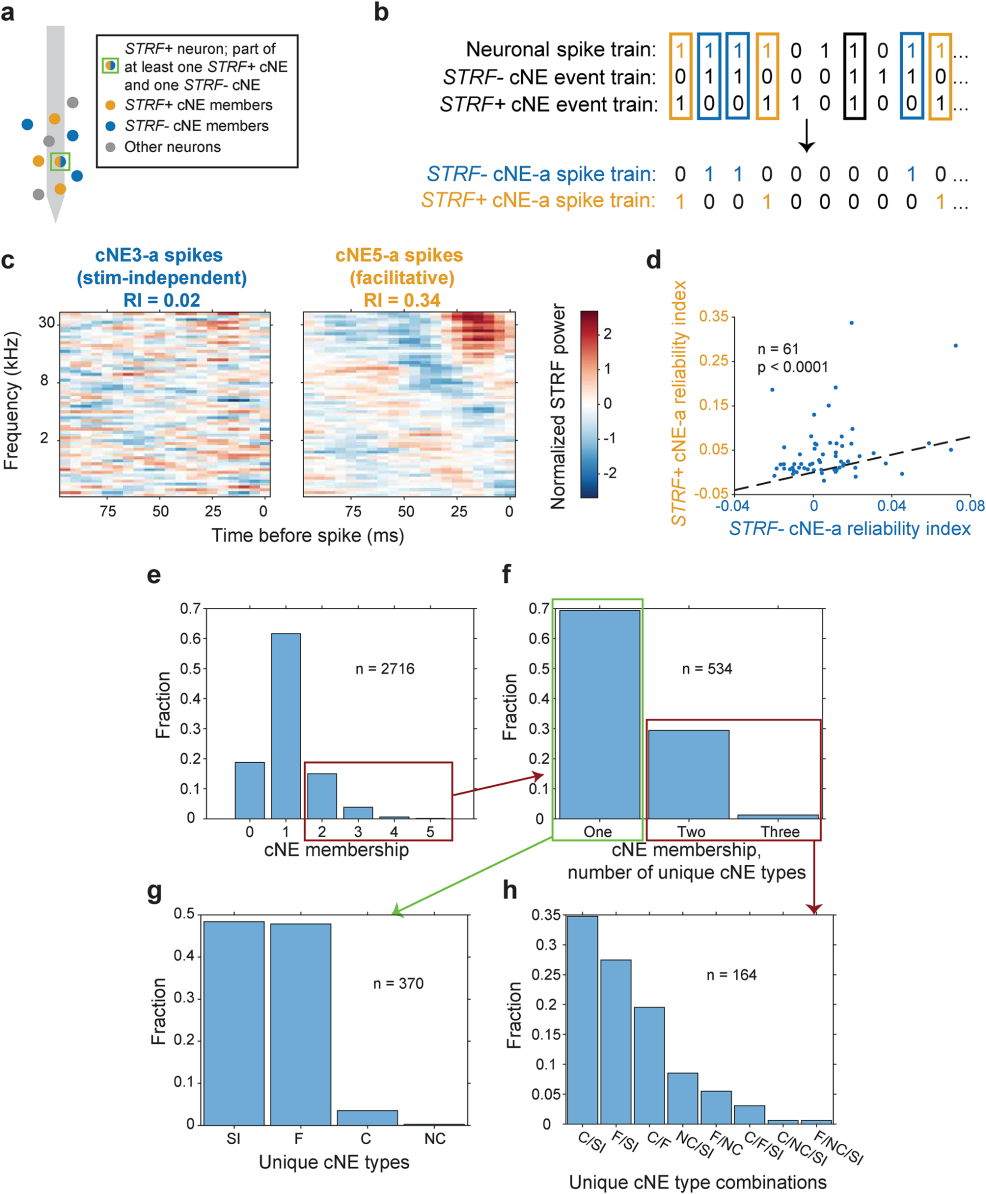
STRFs generated by neuronal spikes from the same neuron associated with *STRF−* cNEs are less stimulus-dependent than those associated with *STRF*+ cNEs. (**a**) Schematic of a sample neuron (green square) that is part of at least one *STRF*+ cNE and one *STRF−* cNE. (**b**) Illustration of a neuronal spike train split into two cNE-a subsets. Blue: spikes associated with the *STRF−* cNE; orange: spikes associated with the *STRF*+ cNE; spikes associated with both cNEs (black rectangle) or neither of the cNEs are omitted. (**c**) Sample STRFs generated from neuronal spikes associated with a stimulus-independent cNE and a facilitative cNE. Spike trains were sub-sampled to get equal numbers of spikes between the two subsets, and the STRF power was normalized. The stimulus-independent cNE-associated spike train was *STRF−* while the facilitative cNE-associated spike train was *STRF*+, despite the fact that the spikes originated from the same neuron. (**d**) RI for *STRF*+ cNE-a spikes was significantly higher than that for *STRF−* cNE-a spikes across the entire population (≈ 79% above black dashed unity line). (**e**) Histogram of cNE membership for all recorded neurons. ≈ 20% of all recorded neurons were part of 2 or more cNEs. The red box indicates the total number of neurons represented in (**f**). (**f**) Histogram of unique categories of cNE membership for all neurons that are part of 2 or more cNEs. ≈ 31% of all neurons that were part of two or more cNEs (≈ 6% of all recorded neurons) were also part of two or more different categories of cNE, while the other ≈ 69% were part of only one type of cNE (≈ 14% of all recorded neurons). The green box represents the total number of neurons represented in (**g**) and the red box represents the total number of neurons represented in (**h**). (**g**) Histogram of cNE categories for neurons that were part of two or more cNEs of the same category. (**h**) Histogram of cNE category combinations for neurons that were part of two or more unique categories of cNEs. (**g**, **h**) *SI* stimulus-independent, *F* facilitative, *C* constructive, *NC* non-constructive. (**d**) Paired t-test.

Leveraging our cNE categories we hypothesized that neurons that contributed to both *STRF*+ cNEs (facilitative or constructive) and *STRF−* cNEs (stimulus-independent or non-constructive) had subsets of spikes that were *STRF*+ and *STRF−,* respectively, based on instantaneous association with either *STRF*+ or *STRF−* cNE events (Fig. 8a,b). As expected, spikes associated with *STRF−* cNE events were also *STRF−* while spikes associated with *STRF*+ cNE events were *STRF*+, despite the fact that both sets of spikes came from the same neuron (Fig. 8c). This difference is also reflected in the RI, with the *STRF*+ spikes associated with *STRF*+ cNE events having significantly greater RI than that of the *STRF−* spikes associated with *STRF−* cNE events (Wilcoxon signed-ranked test, p < 0.0001; Fig. 8d). This confirms that individual spikes of a neuron that are associated with different cNEs can not only differ in their spectro-temporal encoding (Fig. 7), but can also differ in whether they encode specific spectro-temporal features or not.

Across the entire population of recorded neurons (n = 2716), ~ 20% of neurons were part of 2 or more cNEs (Fig. 8e). Out of those neurons (n = 534), ~ 69% took part in cNEs of the same category, while ~ 31% participated in two or three distinct categories of cNEs (Fig. 8f–h). For neurons participating in only one type of cNE, the cNE type was about equally distributed between stimulus-independent and facilitative cNEs (Fig. 8g). For neurons that participated in multiple unique types of cNEs (Fig. 8h), almost all combinations of cNE types were observed, with the most common combination being stimulus-independent cNEs and either constructive or facilitative cNEs (~ 62%).

Taken together, individual spikes from a single neuron that are associated with different cNEs can vary greatly with regards to the nature of what is conveyed about the stimulus ranging from highly specific frequency and envelope information to other, unspecific and currently unknown aspects that may not be linked to obvious stimulus attributes. This implies that STRFs derived from the full spike train of a neuron only represent an approximate, averaged estimate of a neuron’s stimulus preference. In contrast to *STRF*+ cNEs with their low noise, highly selective, and reliable encoding of spectro-temporal stimulus information, single neurons convey, in an interspersed way, several different stimulus-specific or -unspecific attributes.

## Discussion

The aim of this study was to characterize functional properties of cNEs in the cortical column and to enhance our understanding of potential biological interpretations of these functional, multi-neuronal constructs that exhibit enhanced information processing capabilities in AI^7^. To achieve this, we classified cNEs and their associated neurons based on their ability to encode specific acoustic features as reflected in their STRFs. We found that about half of cNEs had STRFs with high specificity to acoustic features (*STRF*+) with the remaining cNEs showing seemingly feature-unspecific activity (*STRF−*). The observation that a given neuron can be associated with several cNEs enabled us to identify distinct subsets of spikes for a given neuron based on their association with different, stimulus-specific or unspecific cNE events. This revealed that an AI neuron can generate functionally heterogenous, intermixed sets of spikes that encode markedly different pieces of information in the presence of an ongoing stimulus. The findings support the notion that cNEs can be considered a fundamental computational unit with events encoding a specific type of information. In contrast, individual neurons, while being a fundamental biological unit of the nervous system, have spike trains that can convey, through multiplexing, several pieces of stimulus-associated and/or stimulus-independent information.

### Classification of cNEs

In this study, we classified cNEs into four distinct groups (facilitative, constructive, stimulus-independent and non-constructive) based on whether the cNEs were *STRF*+ or *STRF−* and the proportion of *STRF*+ member neurons (Fig. 3). While there were functional and physical differences between the four cNE types classified in this manner (Fig. 3c–g), the 50% cutoff for the proportion of *STRF*+ member neurons was ultimately arbitrary and done for the convenience and statistical power of data analysis. In the broader schema, there are two general types of cNEs—stimulus information-enhancing cNEs (facilitative and constructive cNEs) and stimulus-agnostic cNEs (stimulus-independent and non-constructive cNEs) as differentiated by whether the cNE is *STRF*+ or *STRF−*.

The observation that ~ 56% of cNEs were *STRF−* suggests that their prevalence is comparable to that of *STRF−* neurons, which make up ~ 62% of all recorded neurons. It should be noted that the lack of a structured STRF does not mean that the neuron/cNE is not responsive to some kind of information related to or conveyed by the stimulus. This is especially true given the fact that multi-unit, pure-tone mapping at recorded sites showed that multi-unit activity at these sites produced well-defined tuning curves. The high percentage of cNEs without a clear relation to acoustic stimulus features indicates a strong contribution of cortical activity that may relate to other aspects of cortical processing, including stimulus or behavioral context and other top-down influences, such as decision making, memory, and prediction^19,21,31^.

Single neuron recordings in previous studies have pointed to the frequent encounter of unresponsive neurons, at least with regard to modulations in firing rate by relatively simple sound attributes^17,18,32^. The common occurrence of stimulus-unspecific cNEs indicates that these ’unresponsive’ neurons may not represent random activity but are predominately associated with *STRF−* cNEs that can carry biologically relevant information because the statistical probability that member neurons of a cNE fire together by chance is exceedingly small^7^.

### Heterogeneity of neuronal spikes and the efficacy of neuronal coding

Based on temporal association of individual neuronal spikes to cNE events, we were able to assign spikes in a given neuron to subgroups that encode different types and degrees of information (Figs. 6, 7, 8). The ability to functionally tag individual spikes in a train from a single neuron reveals that a neuron can virtually simultaneously be associated with different spectro-temporal stimulus features as well as with spectro-temporally unspecified processing aspects. The idea of multiple neuronal codes is not new, and several studies have shown that this can take place in terms of the coding of different stimulus features^33,34^, different temporal scales of a stimulus^35,36^, or different types of coding, e.g., rate coding vs. temporal coding^37,38^, or spikes vs. local field potentials^39,40^. What we were able to show here appears to be a different type of multiplexing, one at a much finer scale, where individual spikes could encode different types of information regarding a stimulus and representation of the stimulus (STRFs) using the same currency (neural spikes) but in the context of coordinated network activity (cNE events). The cNEs, made up of neurons that are significantly more synchronous with one another than other simultaneously recorded non-members, also distinguishes this study from other recent studies that look at coding of population-level assemblies of indiscriminately pooled neurons over much larger windows of synchrony^41,42^. While the overarching theme from these studies are similar, in that the understanding of the whole (multi-neuronal interactions) provides much more information about neuronal encoding than the sum of its parts (single neurons), it remains to be seen how cNEs defined at such tight windows of synchrony would scale up to thousands of neurons, and will be an interesting avenue of future research.

Spike-to-spike multiplexing can increase the encoding capacity of neuronal responses and efficient representation of distinct stimulus aspects, as supported by theoretical and modeling studies^43,44^. This, however, renders the average spike-associated information for single neurons inherently unreliable for identifying what stimulus features are encoded over the whole duration of the stimulus. By contrast, cNE events appear to encode only one type of these conceptually different stimulus information aspects by reading out the jointly associated events from several neurons. Taken together, these observations reinforce the idea that cNEs, due to their higher information specificity, should be considered the principal computational unit of information processing in AI.

### Relation of spike heterogeneity to arousal and behavioral training

The functional heterogeneity of neuronal spikes in AI has likely behavioral implications. The attentional state of an animal can cause rapid changes in a neuron’s receptive field^45–47^. This effect is reversible when an animal stops attending to the task, or is forced to change its decision-making goal, and has been referred to as “rapid plasticity” in AI^45–47^. This could be due to changes in the functional heterogeneity of spikes as supported by theoretical and modeling studies^43,48,49^. Since neurons can adopt somewhat different STRF attributes when associated with different cNEs (Fig. 7), rapid changes in the dominant receptive field occurs by engagement in a detection or discrimination task that biases a neuron’s activity toward the most useful cNE for performing the task, potentially selected for via top-down modulations. Hence, rapid plasticity^45–47^ could be interpreted as a reinforcement of network configurations already in place.

Taken together, we postulate that auditory cortical processing is based on (1) neurons with functionally heterogeneous spikes and (2) local networks or cNEs with functionally homogeneous events focusing on one of several different types of stimulus-associated information. We postulate that all AI neurons participate in multiple cNEs and that each neuron would be involved in encoding a mixture of spectro-temporally specific and unspecific stimulus aspects. The nature of the unspecific stimulus aspects still needs to be determined, e.g., while animals are engaged in tasks or are in different levels of arousal. Identifying the different types of information encoded by cNEs would elucidate the roles of different sensory processing aspects for efficient information transmission and integration between top-down cognitive areas and bottom-up auditory areas^19–21,31^.

## Methods

### Animals

All experimental procedures were approved by the Institutional Animal Care and Use Committee (IACUC) at the University of California San Francisco and were carried out in accordance with NIH guidelines. 17 female wild type Sprague-Dawley rats (200–300 g, 2–3 months old; RRID: MGI: 5651135), sourced from Charles River, were used in this study. All methods were carried out in accordance with relevant guidelines and regulations in compliance with the ARRIVE guidelines (https://arriveguidelines.org).

### Electrophysiology

The rats were anesthetized with a mixture of ketamine and xylazine. The primary auditory cortex (AI) was mapped by using multi-unit responses to pure tones of different frequencies (1–40 kHz) and intensities (10–80 dB). Regions with short latencies (10–30 ms) and a tonotopic gradient in the rostrocaudal axis were identified to be AI^50^. Recordings were made using either one of three 32-channel probes (a1 × 32-poly1, a1 × 32-poly2, a1 × 32-poly3; NeuroNexus), or a 64-channel probe (H3; Cambridge NeuroTech), inserted perpendicular to the cortical surface to a depth of approximately 800–1400 µm using a microdrive (David Kopf Instruments). Neural traces were band-pass filtered between 500 and 6000 Hz and were recorded to disk at 20 kHz sampling rate with an Intan RHD2132 Amplifier system. Traces were spike-sorted offline using MountainSort, a fully automated spike sorter^51^.

### Stimulus

All stimulus generation, data and statistical analyses were performed with MATLAB (Mathworks). All details on standard hypothesis tests used in individual figures can be found in the figure legends and main text.

The stimulus was either a dynamic moving ripple or a ripple noise^7,23^. The dynamic moving ripple (DMR) was a temporally varying broadband sound (500 Hz–40 kHz) made up of approximately 50 sinusoidal carriers per octave, each with randomized phase. The maximum spectral modulation frequency of the DMR was 4 cycles/oct, and the maximum temporal modulation frequency was 40 cycles/s. The maximum modulation depth of the spectro-temporal envelope was 40 dB. Mean intensity was set at 30–50 dB above the average pure tone threshold within a penetration. The ripple noise (RN) stimulus was the sum of 16 independently created DMRs. Both DMR and RN were presented as long and continuous 10-, 20- or 30-min stimuli. Spectro-temporal receptive fields (STRFs) were calculated by spike triggered averaging of the DMR and RN stimuli (Supplementary Fig. 1a).

### Coordinated neuronal ensemble (cNE) detection algorithm

Complete details of the cNE detection algorithm used in this study are described in a previous study^7^. The cNE detection algorithm used in this study was based on dimensionality reduction techniques^25^. Principal component analysis (PCA) was applied to the z-scored spike matrix (10-ms bins) to obtain an eigenvalue spectrum. Eigenvalues that exceeded the upper bounds of the Marčenko–Pastur distribution^52^ were deemed significant and represented the number of detected cNEs. The eigenvectors corresponding to significant eigenvalues were then processed using independent component analysis (ICA). The resulting independent components (ICs) represent the contribution of each neuron to each cNE. The activity of each cNE was calculated by then projecting the z-scored spike matrix onto the ICs^25^. cNE membership and binarized cNE activity were determined using surrogate methods, which are detailed in a previous study^7^.

### Reliability index (RI) of STRF and CRH

A reliability index (RI) metric, adapted from^24^, was used to quantify the strength of STRFs and conditioned response histograms (CRHs) from both neurons and cNEs. To calculate RI, all spike or cNE event trains were divided into 1-min segments, and the STRF/CRH of each 1-min segment was calculated. The set of 1-min segments was split into two equal groups (segments A and B) and two STRFs/CRHs (STRFs A and B) were obtained by averaging the STRFs/CRHs from each of the corresponding sets of 1-min segments. The similarity of STRF/CRH A and B was then calculated using Pearson’s correlation. This STRF/CRH similarity was calculated over 100 iterations, with different combinations of 1-min segments for each iteration. RI was then calculated as the mean STRF/CRH similarity over the 100 iterations (Supplementary Fig. 1b).

To determine the significance of each STRF/CRH, each corresponding spike or cNE event train was circularly shuffled by increasing multiples of N/100, where N is the number of time bins for each spike or cNE event train, 100 times. For each shuffle, 100 STRF/CRH similarity values were calculated as described above, giving rise to 100,000 STRF/CRH similarity values, which formed the null distribution of RI. A p-value was calculated by quantifying the proportion of the total distribution (null distribution and real RI value) that was larger than or equal to the real RI value. STRFs/CRHs with p < 0.05 were considered significant, while STRFs/CRHs with p ≥ 0.05 were not significant (Supplementary Fig. 1c).

For all direct comparisons of RI between two different groups (Figs. 3e–h, 6d,e, 8d), the group with the larger number of spikes or events was subsampled to match the number of spikes or events of the smaller group. Each random subsampling was done 20 times, and the mean RI of the 20 iterations was used as the RI value for comparison.

### Pairwise cross-correlation sharpness

The pairwise cross-correlation sharpness measure (Fig. 3f, Supplementary Fig. 4e) was calculated based on a previous study^7^. For each sharpness value, a pairwise cross-correlation function between two spike or cNE event trains was calculated with a time resolution of 0.5 ms and a maximum delay of 50 ms. The peak delay for each pairwise cross-correlation function was estimated and each function was folded around that delay, to a maximum of 20 ms. Sharpness was then estimated by the delay (in ms) that accounts for half the spike count in each folded cross-correlation function. For an illustration of this calculation, see previous study^7^.

### Calculation and comparison of phase-locking indices (PLIs)

Phase-locking indices (PLIs) were calculated using raw, unsorted multi-unit activity recorded during spontaneous epochs (i.e. no stimulus was presented). PSTHs were calculated by summing all MUA activity across all channels, and power spectrums were calculated for each penetration. The PSTHs were then bandpass filtered by f ± 0.3 Hz, where f is the frequency with the peak power. The instantaneous phase of each signal was then calculated using Hilbert transform. The instantaneous phase for each penetration was then used to generate phase histograms for each cNE, based on the instantaneous association between significant cNE activity and the instantaneous phase. PLIs for each cNE were then obtained by calculating the magnitude of the mean phase vector of each phase histogram.

Whether a cNE was *STRF*+ or *STRF−* was determined by STRF properties obtained from the contiguous stimulus-evoked responses, and PLIs between those two categories were compared in Fig. 5d.

## Supplementary Information

Supplementary Figures.

## Data Availability

The datasets generated during and/or analyzed during the current study are available from the corresponding author on reasonable request.
