# Supplementary material for: Information diversity in individual auditory cortical neurons is associated with functionally distinct coordinated neuronal ensembles
Source: Sci Rep. 2021 Feb 18;11:4064. doi: 10.1038/s41598-021-83565-7 (PMC7893178; doi:10.1038/s41598-021-83565-7)
Supplement: Supplementary file 1 — Supplementary Figures. [file 41598_2021_83565_MOESM1_ESM.pdf]

Supplementary Figures and Legends

Information Diversity in Individual Auditory Cortical Neurons is Associated with Functionally Distinct Coordinated Neuronal Ensembles

Jermyn Z. See[1,2], Natsumi Y. Homma[1,2], Craig A. Atencio[1,2], Vikaas S. Sohal[1,3], Christoph E. Schreiner[1,2,4]*

[1]Weill Institute for Neuroscience, Kavli Institute for Fundamental Neuroscience, and Sloan-Swartz Center for Theoretical Neurobiology, University of California, San Francisco, 675 Nelson Rising Lane, San Francisco, CA 94158-0444, USA

[2]Coleman Memorial Laboratory, Department of Otolaryngology – Head and Neck Surgery

[3]Department of Psychiatry

[4]Lead Contact

*Corresponding author: chris@phy.ucsf.edu

Supplementary Figure 1

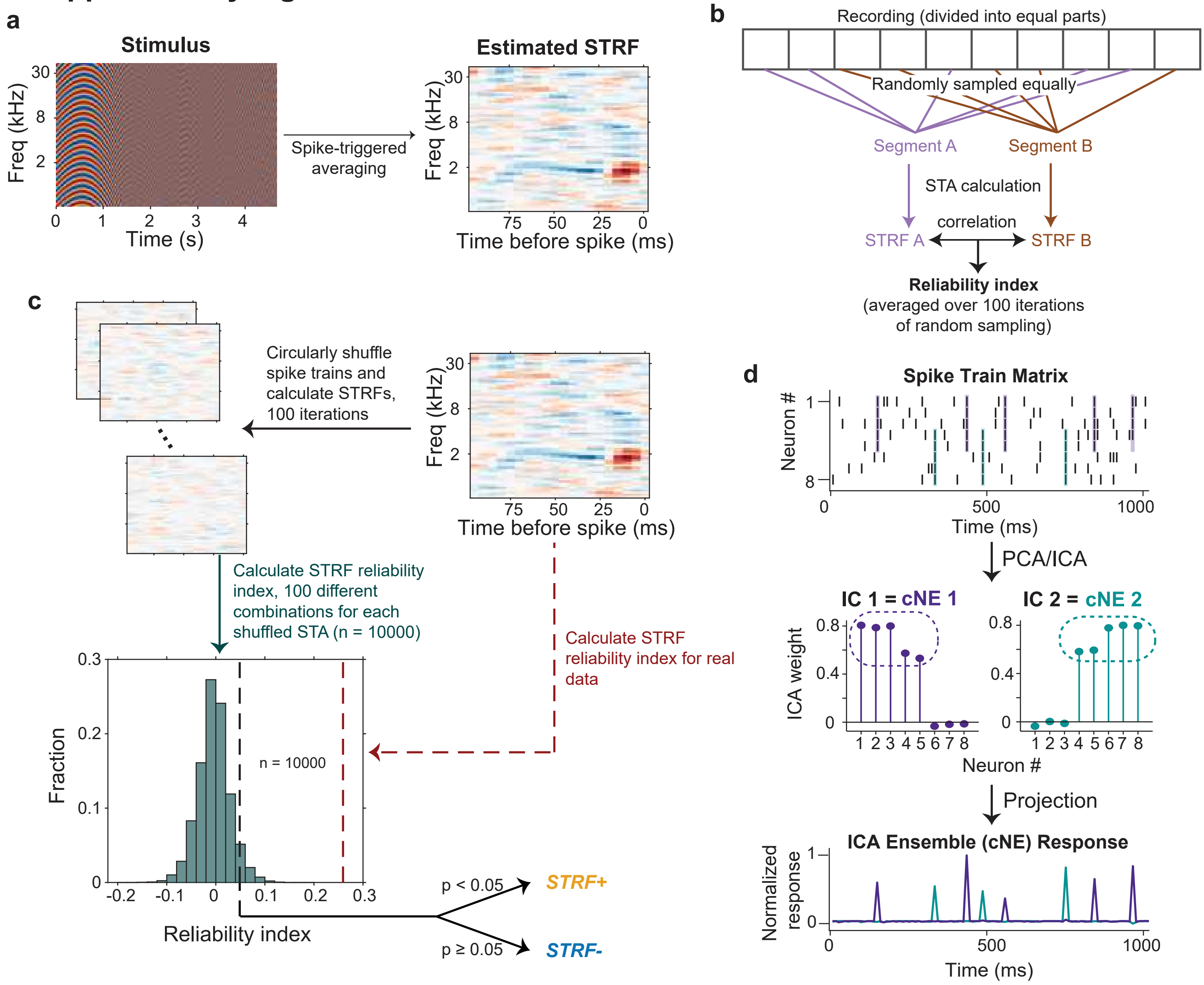

Supplementary Figure 2

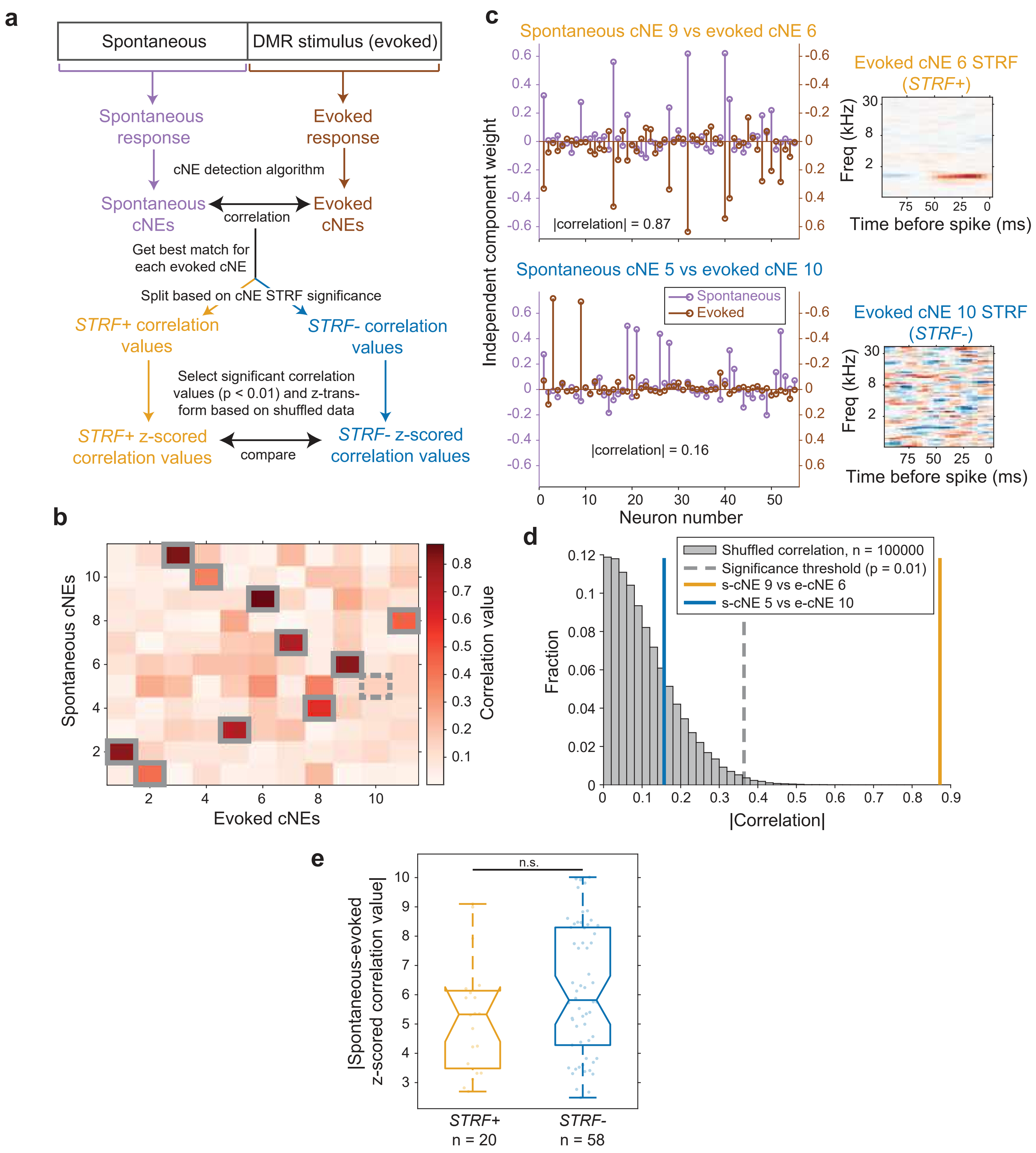

Supplementary Figure 3

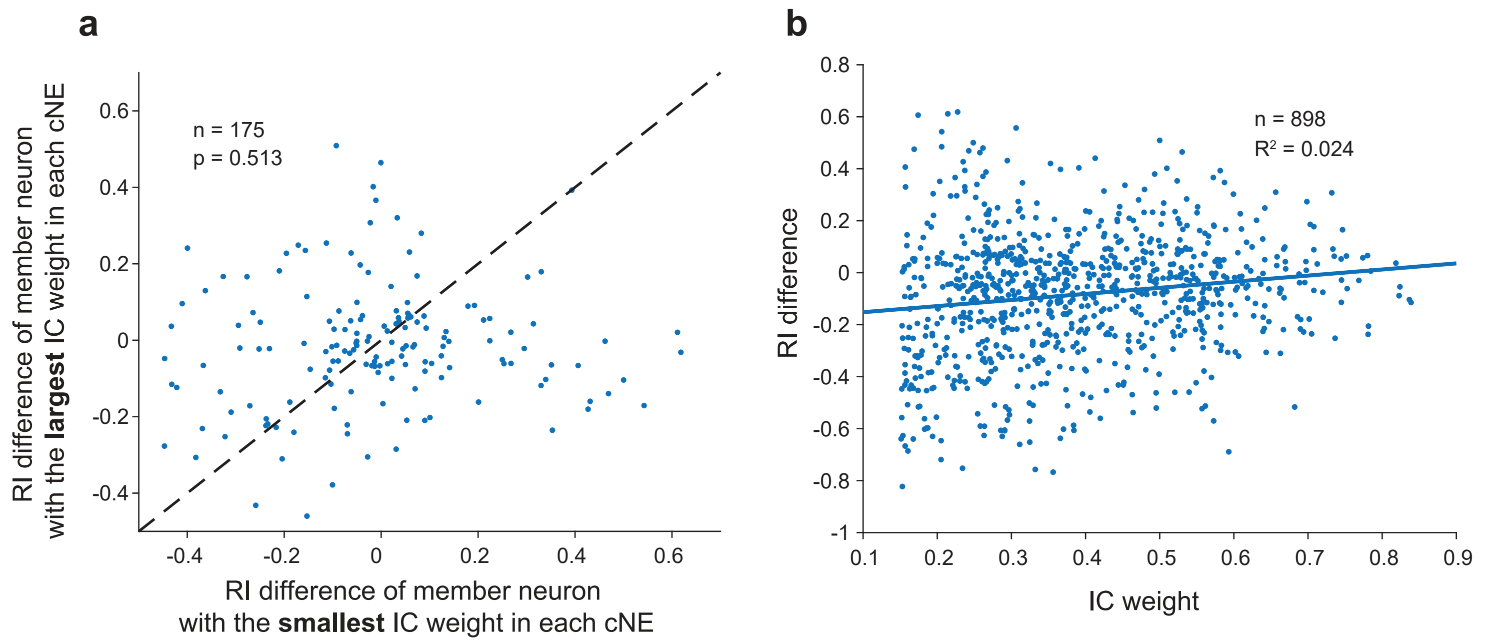

Supplementary Figure 4

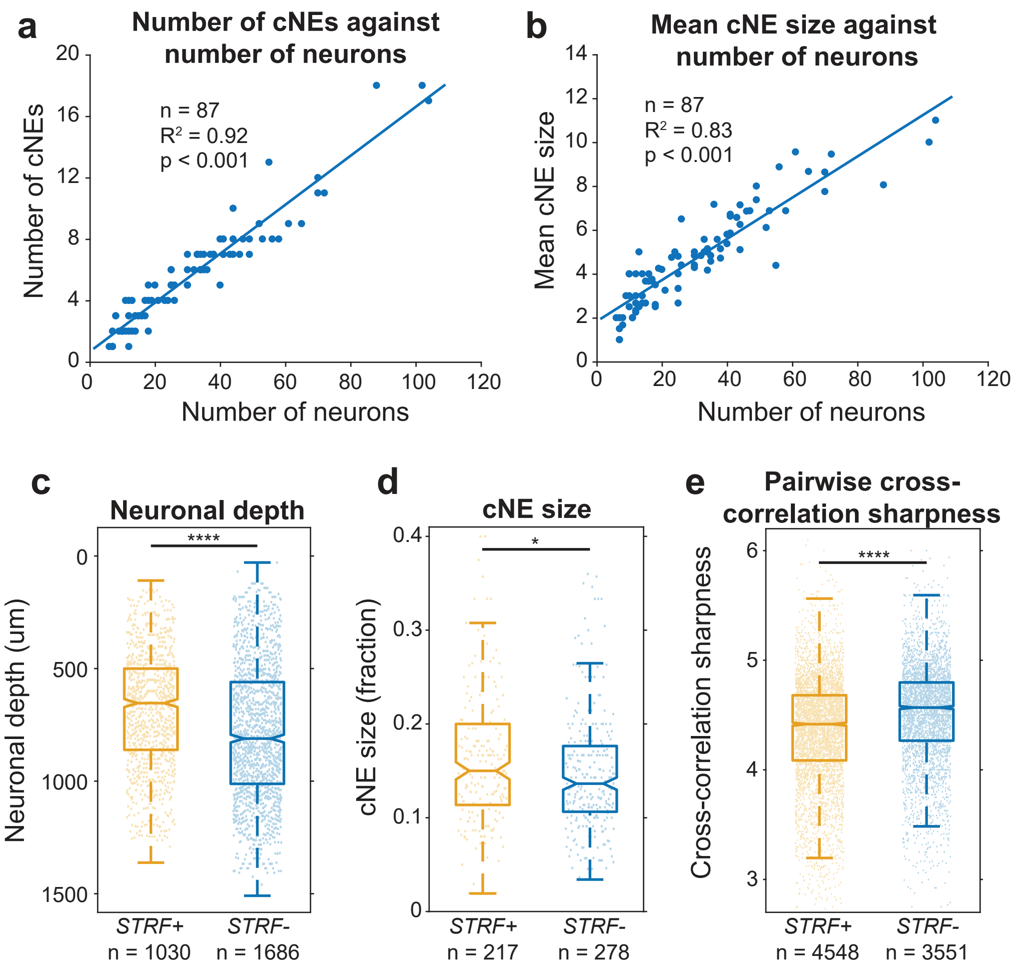

Supplementary Figure 5

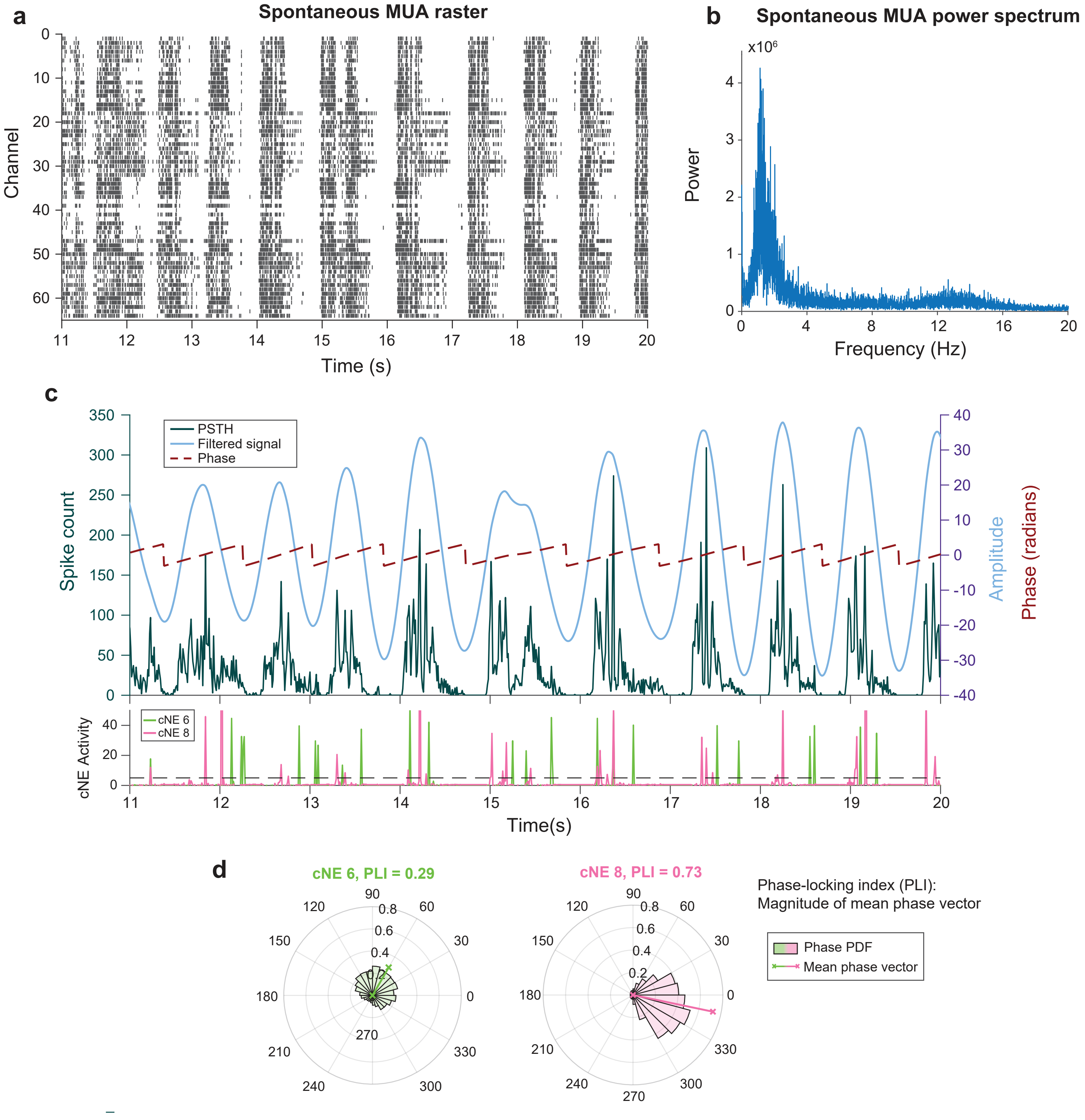

Supplementary Figure 6

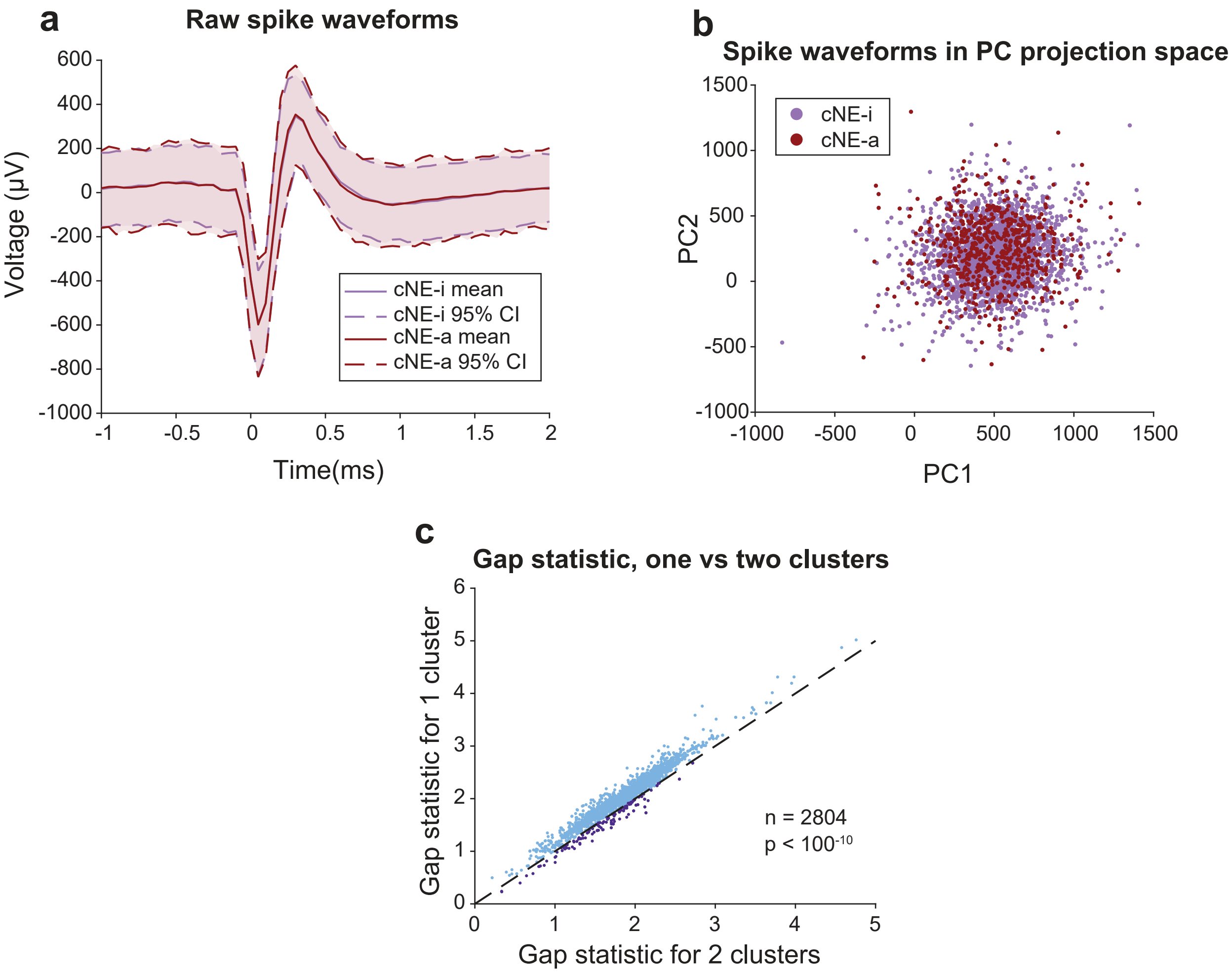

Supplementary Figure 7

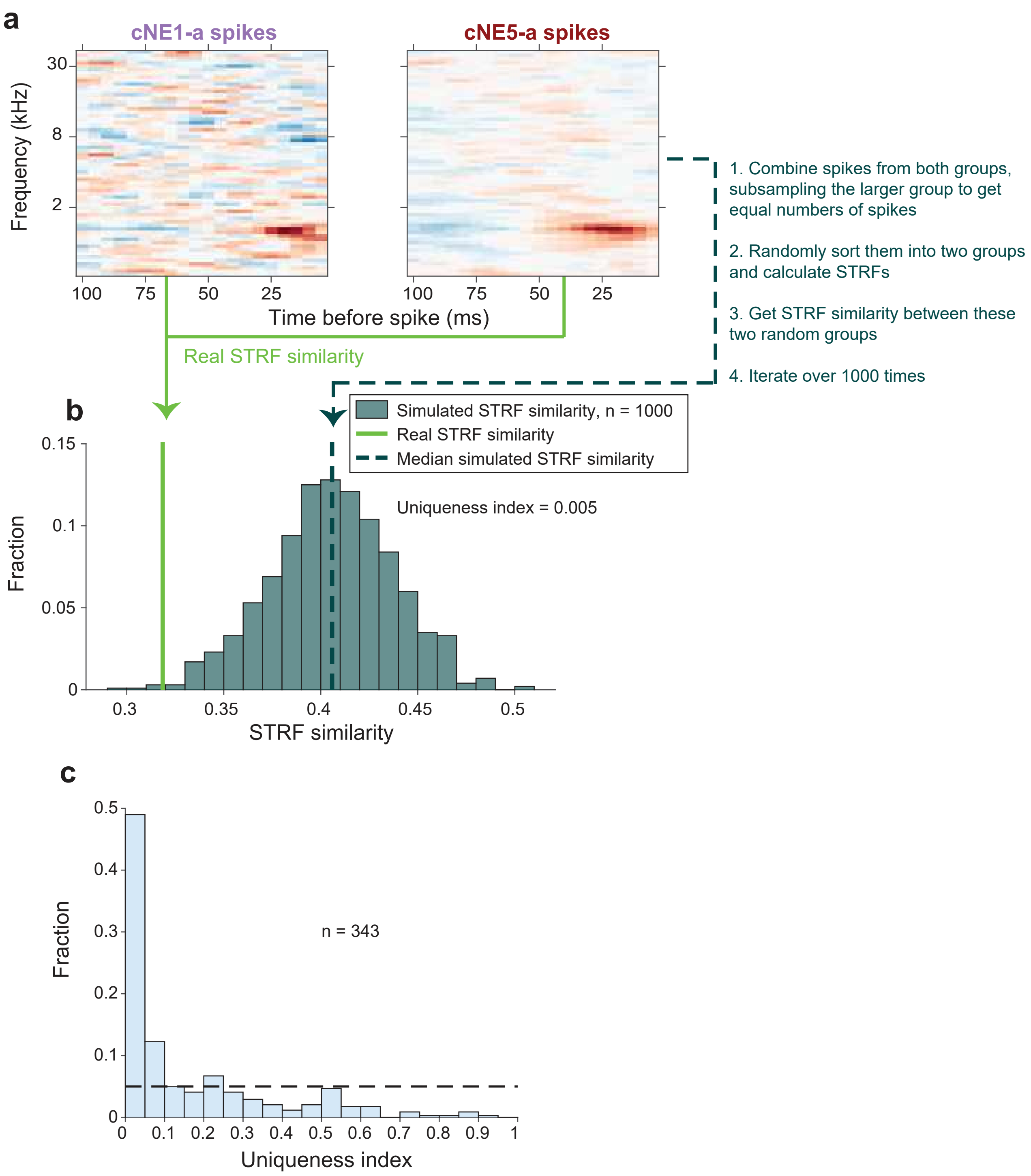

Supplementary Figure 8

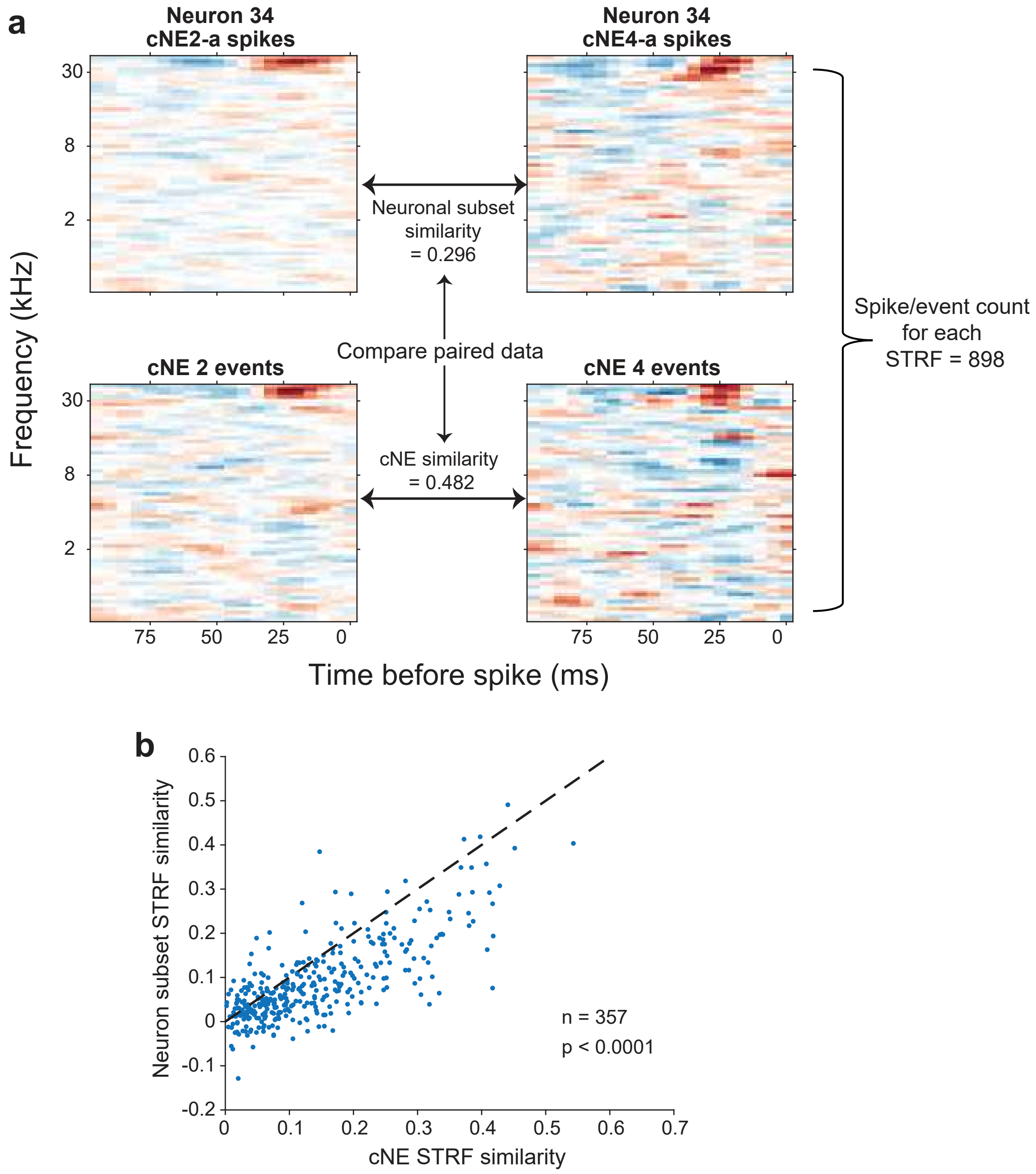

Supplementary Figure Legends

Supplementary Figure 1. Main experimental and analytical methods. (a) Stimulus sample and derived receptive field. (left) Spectrogram of the dynamic moving ripple stimulus, a temporally varying broadband sound made up of approximately 50 sinusoidal carriers per octave. (right) Spectral-temporal receptive field (STRF), calculated by spike triggered averaging. **(b)** Calculation of the RI to determine if a STRF was significant (*STRF+*). Each spike or event train was split up into two random but equal groups and had their STRFs calculated. The RI is the average of the similarity between each pair of STRFs over 100 iterations. **(c)** Method of determining the significance of an STRF. Each spike train was circularly shuffled randomly over 100 iterations to generate 100 pairs of null STRFs. Every possible pairwise combination for STRF similarity was calculated to generated a null distribution (dark green bars) and the null distribution was compared against the real STRF similarity calculated in **(b)** to get a p-value. A STRF is *STRF+* if $p < 0.05$. Otherwise, it is *STRF-*. **(d)** Toy model illustrating the detection method for cNEs (see Methods). (top) A spike train matrix is binned, z-scored. Neurons 1 – 5 and neurons 4 – 8 were modeled to have a high probability of coordinated activity. (middle) The spike trains were processed with principal component analysis and then independent component analysis to determine cNE membership. IC 1 and 2 identify neurons 1 – 5 and neurons 4 – 8 as cNEs respectively. (bottom) The ICs were projected back onto the z-scored spike train matrix to determine cNE activity, and these peaks match the periods of coordinated activity seen in the spike matrix.

Supplementary Figure 2. *STRF+* or *STRF-* cNEs were equally stable in spontaneous activity. (a) Illustration of the method used to compare *STRF+* and *STRF-* cNEs with respect to preservation

of cNE identity between spontaneous and evoked activity. **(b)** Sample correlation matrix of the similarity of cNEs (based on calculated independent components) identified in successive spontaneous and evoked epochs from one penetration. Best matches between evoked and spontaneous cNEs were determined by the highest value in each column. Significant matches were marked in solid gray rectangles while non-significant matches were marked in dashed gray rectangles. **(c)** Example of two pairs of matches between spontaneous and evoked cNEs. (upper panels) Sample of an evoked *STRF+* cNE and high similarity between spontaneous and evoked cNEs. (lower panels) Sample of an evoked *STRF-* cNE and low similarity between spontaneous and evoked cNEs. **(d)** Determination of significant matches between spontaneous and evoked cNEs, with threshold set at p = 0.01 (gray dashed line). The *STRF+* cNE in **(c)** was also significantly matched to its corresponding spontaneous cNE (orange line). The *STRF-* cNE in **(c)** was not a significant match to its corresponding spontaneous cNE (blue line). **(e)** Correlation values were z-scored based on their corresponding null distributions and compared. Only cNEs with significant matches, as calculated in **(d)** were included. There was no significant difference between *STRF+* cNEs and *STRF-* cNEs. **(e)** Mann-Whitney U test.

Supplementary Figure 3. STRFs of facilitative cNEs are not dominated by member neurons with the highest RI. (a) Difference in RI between a facilitative cNE and the *STRF+* member neuron with the highest absolute IC weight vs difference in RI between a facilitative cNE and the *STRF+* member neuron with the lowest (but still significant) absolute IC weight. There was no significant disparity in the RI difference between *STRF+* neurons with the highest and lowest IC weights. **(b)** Scatter plot of RI difference against absolute IC weight of *STRF+* member

neurons. This covariance only accounted for about 0.02 of the variance seen in the data. **(a)** Paired t-test.

Supplementary Figure 4. Statistics of *STRF+* and *STRF-* cNEs and neurons. (a) The number of cNEs identified is positively correlated with the number of neurons recorded. **(b)** The mean cNE size is also positively correlated with the number of neurons recorded. **(c)** *STRF+* neurons were found at a shallower depth than that of *STRF-* neurons. **(d)** *STRF+* cNEs made up a larger proportion of recorded neurons than *STRF-* cNEs. **(e)** Cross-correlation functions between neuronal members of *STRF+* cNEs were sharper than that of *STRF-* cNEs. *$p < 0.05$, ****$p < 0.0001$, Mann-Whitney U test.

Supplementary Figure 5. Illustration of PLI calculation. (a) Raster plot for spontaneous activity of multi-unit activity (MUA) in one example penetration. **(b)** Power spectrum of multi-unit activity. **(c)** (top) MUA was summed across all channels to get a population PSTH (dark green solid line). The PSTHs were then bandpass-filtered based on f ± 0.3 Hz, where f is the frequency with the highest power, to get a filtered signal (light blue solid line). The instantaneous phase was then calculated using Hilbert transform (brown dashed line). (bottom) The instantaneous phase was then compared with cNE activity to generate the phase histograms in **(d)**. **(d)** Phase histograms of the two example cNEs in **(c)**. The phase-locking index (PLI) is calculated as the magnitude of the mean phase vector.

Supplementary Figure 6. cNE-i and cNE-a spike waveforms cannot be distinguished from each other. (a) Raw spike waveforms of cNE-i and cNE-a spikes from a sample neuron. **(b)** Spike waveforms of neuron in **(a)** in two-dimensional PC projection space. cNE-i spikes are labeled in

purple and cNE-a spikes in red. **(c)** Gap statistic[51] for clustering, which determines the ideal number of clusters from the data points in two-dimensional PC space. The gap statistic significantly favors a single cluster across the entire population of neurons that belong to at least one cNE. Neurons that had a higher gap statistic for 2 clusters (~5%) were omitted from the analyses in Figs. 6 - 8.

Supplementary Figure 7. Statistical method for quantifying the difference between STRFs generated by cNE-a subsets of spikes from the same neuron. (a) Sample of two cNE-a subset STRFs from a *STRF+* neuron that is a member of at least two *STRF+* cNEs. The method for generating a null distribution of STRF similarity is described in dark green. **(b)** Histogram of simulated STRF similarity, with the median represented by the dark green dashed line. The STRF similarity between the two STRFs in **(a)** is represented by the bright green solid line. The uniqueness index is the proportion of the entire distribution (dark green bars and bright green solid line; n = 1001) that is smaller than or equal to the real STRF similarity value (bright green solid line). Hence, the minimum possible uniqueness index ≈ 0.001. **(c)** Histogram of uniqueness index values across all neurons shared between multiple significant cNEs. Almost 50% of such neurons had uniqueness index values of < 0.05.

Supplementary Figure 8. Difference in STRFs between neuronal subsets determined by two cNEs cannot be trivially explained by the difference in STRFs between the same cNEs. (a) Illustration of the comparison between neuronal subset STRF similarity and its corresponding cNE STRF similarity. Spike/event counts for each spike/event train that generated the STRFs were normalized to have the same number of spikes/events. In this example, the STRF similarity between the neuronal subsets is lower than that between the cNEs. **(b)** Comparison of STRF

similarity across the whole population of shared neurons. cNE STRF similarity was significantly higher than that of neuronal subset STRF similarity (≈80% below black dashed unity line). **(b)** Paired t-test.
